# Potential Serum Biomarkers for Postoperative Neurocognitive Disorders Based on Proteomic Analysis of Cognitive-Related Brain Regions

**DOI:** 10.3389/fnagi.2021.741263

**Published:** 2021-09-29

**Authors:** Yitong Li, Lei Chen, Zhengqian Li, Yanan Song, Yi Yuan, Taotao Liu, Jingshu Hong, Qian Wang, Huixian Chang, Zhongshen Kuang, Jindan He, Yue Li, Xinning Mi, Dengyang Han, Ning Yang, Xiangyang Guo

**Affiliations:** ^1^Department of Anesthesiology, Peking University Third Hospital, Beijing, China; ^2^Department of Anesthesiology, Beijing Jishuitan Hospital, Beijing, China; ^3^School of Information Science and Engineering, Yanshan University, Qinhuangdao, China

**Keywords:** postoperative neurocognitive disorders, proteomic analysis, serum, potential biomarker, geriatric (aging), 14-3-3β/α, alpha-2 macroglobulin (α2-macroglobulin), caseinolytic protease

## Abstract

Postoperative neurocognitive disorders (po-NCD), including postoperative delirium (POD) and delayed neurocognitive recovery (dNCR), are common in geriatric surgical patients. However, the ideal diagnostic biomarkers to predict individual risks of po-NCDs have not been identified. In this study, proteomic analysis was used to detect dysregulated proteins in three cognitive-related brain regions, the hippocampus, prefrontal cortex, and temporal lobe, of aged dNCR rats. The common affected proteins in these three brain regions were further verified by real-time polymerase chain reaction and western blotting. Furthermore, serum samples from aged rats with dNCR and elderly hip fracture patients with POD were also assessed with enzyme linked immunosorbent assays to investigate the biomarker potential of these dysregulated proteins. The increased expression levels of haptoglobin, caseinolytic protease (ClpP), and alpha-2 macroglobulin (A2M) as well as decreased expression levels of 14-3-3β/α and biliverdin reductase-A (BVR-A) were validated by proteomic analysis in the hippocampus, prefrontal cortex, and temporal lobe of aged dNCR rats. The increased expression of haptoglobin and decreased expression of 14-3-3β/α were further demonstrated in the three brain regions by western blotting. Moreover, increased levels of S100A6 and BVR-A in the hippocampus, S100A6 in the prefrontal cortex, and A2M in the temporal lobe were also observed. More intriguingly, both decreased serum 14-3-3β/α and increased A2M in geriatric POD patients as well as decreased serum ClpP in aged dNCR rats were verified. These results not only indicate potential diagnostic biomarkers for po-NCD but also provide directions for further pathological investigations.

**Clinical Trial Registration:** www.ClinicalTrials.gov, identifier [ChiCTR1900027393].

## Introduction

Postoperative neurocognitive disorders (po-NCDs), including the acute form termed postoperative delirium (POD) and the long-lasting form termed delayed neurocognitive recovery (dNCR), are the most frequent complications after surgical treatment in the elderly (Eckenhoff et al., 2020). The incidence of po-NCDs ranges from 10 to 65% and varies depending on a variety of factors such as age, sex, education level, comorbidities, and surgery type (Boone et al., 2020). The occurrence of po-NCDs is associated with longer hospital stays, increased hospital and social costs, poorer recovery, and increased morbidity and mortality (Rudolph et al., 2008). However, no reliable biomarkers currently exist to predict and diagnose po-NCDs. Therefore, it is imperative to explore novel and effective biomarkers for po-NCDs (Androsova et al., 2015; Schaefer et al., 2019).

At present, investigations of biomarkers for po-NCDs mainly focus on the underlying causation and mechanisms including genetic risk factors and physiological and immunological changes (Androsova et al., 2015; Schaefer et al., 2019). Our previous research also indicated that alteration of α-synuclein levels in L1CAM-carrying exosomes in the plasma was positively correlated with both the interleukin-6 concentration and POD severity in older patients following hip fracture surgery (Yuan et al., 2020). However, the pathological mechanisms underlying po-NCDs are extremely complicated and not completely understood. Therefore, it may be difficult to identify specific markers from the existing mechanisms, and it may be more advantageous to screen biomarkers using metabolomics and proteomics. We found that 18 metabolites and 33 lipids were dysregulated in the preoperative cerebrospinal fluid (CSF) of POD patients, and among them, phosphatidylethanolamine may be a potential biomarker for POD (Han et al., 2020c). Our other study also revealed 63 dysregulated CSF proteins in older patients with POD following hip fracture surgery, and among them the V-set, transmembrane domain-containing protein 2B, and coagulation factor V may be risk biomarkers (Han et al., 2020b). However, in contrast to peripheral blood sampling, obtaining CSF is not a routine and convenient procedure and cannot be performed in all patients.

po-NCDs are certain to cause pathophysiological changes in cognition-related brain regions. The hippocampus, prefrontal cortex, and temporal lobe are all important brain regions involved in cognitive function. The hippocampus plays a vital role in learning and memory processes (Whitlock et al., 2006), and the prefrontal cortex is important for working memory and self-regulatory and goal-directed behaviors (Miller, 2000). In addition to participating in the memory process, the temporal lobe is also associated with a wide range of cognitive functions, such as attention, vision, executive function, and decision-making ability (Cahn-Weiner et al., 2009; Bora and Meletti, 2016; Gliebus, 2018; Berron et al., 2020). If dysregulated proteins common to the above three brain regions are also verified to be abnormal in the peripheral blood, they could likely be used as convenient diagnostic biomarkers.

In this study, we identified the common altered proteins in three cognitive-related brain regions, the hippocampus, prefrontal cortex, and temporal lobe, by proteomic analysis and then determined their serum concentrations in aged dNCR rats and elderly POD patients. The results from the current study not only identify potential diagnostic biomarkers for po-NCDs but also provide a basis for additional pathological research.

## Materials and Methods

### Animals and Ethics

Aged male Sprague–Dawley rats (20 months old, 550–650 g) were purchased from the Dongchuang Laboratory Animal Center (Changsha, Hunan, China) and housed in a temperature (22 ± 0.5°C) and humidity (55 ± 5%) controlled room with a 12 h light/dark cycle (lights on at 07:00) with *ad libitum* access to food and water. The rats were allowed 2 weeks to acclimate to the new environment before the experimental procedures. The experimental protocol was approved by the Peking University Biomedical Ethics Committee Experimental Animal Ethics Branch (Approval No. LA201413). All efforts were made to minimize the number and suffering of animals. Animals were randomly assigned to the control group and surgery group before anesthesia and surgery.

### Anesthesia and Surgery

The anesthesia and surgery protocol was in accordance with that used in our previous report (Li et al., 2016). Anesthesia induction (4% isoflurane for 3 min) was performed in an anesthetic chamber. Rats were then placed in a supine position on an operating table with a thermal blanket with a constant temperature of 35°C. Rats were then endotracheally intubated and mechanically ventilated with 1.5–2.0% isoflurane and 40% oxygen. The laparotomy was performed aseptically. Briefly, the body region undergoing surgery was shaved and sterilized. A 4-cm vertical incision was made 5 mm below the lower right rib. Approximately 10 cm of the small intestine was exteriorized and vigorously rubbed between the thumb and index finger for 30 s. Then, the small intestine was placed back in the abdominal cavity, and the incision was sutured in layers. At the end of the operation, isoflurane inhalation was stopped. Rats were extubated when spontaneous breathing was restored, and ventilation was ceased when body reactions had recovered. The surgery lasted 20–25 min. For surgical pain relief, 3 mg (1 ml) of oxybuprocaine hydrochloride gel was aseptically applied to each animal before suturing the incision. The mucous color was monitored every 5 min until rats could maintain an upright posture and walk normally. The control group received treatment similar to the surgery group, except laparotomy was not performed.

### Morris Water Maze (MWM) Test

The MWM test (Sunny Instruments Co. Ltd., Beijing, China) was performed to evaluate spatial learning and memory abilities as we previously described, with modifications (Han et al., 2020a). Swimming was recorded with a video tracking system (Sunny Instruments Co. Ltd.). Rats were subjected to four trials (cut off time 120 s) daily for 5 consecutive days before and 2 days after surgery. On the first and second day after surgery, the probe trial test was performed (after the platform was removed, the rat was allowed to swim for 120 s). The escape latency, swimming speed, and target quadrant crossing times of rats were recorded and analyzed.

### Euthanasia and Sample Collection

Euthanasia (6% isoflurane for 60 s) was performed immediately after the MWM was completed. Blood samples (1.0 ml from each rat) were obtained from the inferior vena cava. Then, the hippocampus, prefrontal cortex, and temporal lobe were immediately dissected. Blood samples were stored at 4°C for 1 h until coagulation and then centrifuged (at 3000 × *g* for 10 min) to obtain serum. Both the serum and tissue samples were stored at −80°C until further processing.

### Liquid Chromatography Tandem-Mass Spectrometry (LC-MS/MS) Analyses

Cerebral samples from animals were processed and subjected to protein extraction, reduction, alkylation, and trypsin digestion according to the manufacturer's protocol to prepare filter-aided samples. For each sample, 100 μg of tryptic peptide was used for isobaric tags for relative and absolute quantitation labeling. The labeled samples were then mixed and subjected to Sep-Pak C18 desalting. The mixture was fractionated using high pH reverse phase chromatography and separated into 15 fractions. Each fraction was vacuum-dried and stored at −80°C until mass spectrometry analysis.

The mass spectrometry analysis experiments were carried out on a hybrid quadrupole-time-of-flight LC-MS/MS mass spectrometer (TripleTOF 5,600, SCIEX, Framingham, MA, USA) equipped with a nanospray source. Peptides were first loaded onto a C18 trap column (5 μm, 5 × 0.3 mm, Agilent Technologies, CA, USA) and then eluted into a C18 analytical column (75 × 150 mm, 3 μm particle size, 100 Å pore size, Eksigent, CA, USA) at a constant flow rate of 300 nl/min. Each scan cycle consisted of one full-scan mass spectrum (with m/z ranging from 350 to 1,500, ion accumulation time 250 ms) followed by 40 MS/MS events (m/z ranging from 100 to 1,500, ion accumulation time 50 ms). All samples were analyzed in random order. Raw data from the Triple TOF 5,600 were analyzed with ProteinPilot V4.5 (SCIEX, Framingham, MA, USA) using the Paragon database search algorithm and the integrated false discovery rate analysis function. Spectra files were searched against the UniProt rat reference proteome database using the following parameters: sample type, isobaric tags for relative and absolute quantitation 8plex (peptide labeled); Cys alkylation, iodoacetamide; digestion, trypsin; and quantitate, bias correction, and background correction were enabled for specific processing. The search effort was set to rapid ID. Search results were filtered with an unused score and false discovery rate threshold of 1.3%. Decoy hits were removed, and the remaining identified results were used for further function and pathway analysis.

### RNA Isolation and Real-Time Polymerase Chain Reaction (RT-PCR)

mRNA expression levels of 14-3-3β/α, haptoglobin, S100A6, caseinolytic protease (ClpP), α-2 macroglobulin (A2M), biliverdin reductase-A (BVR-A), and β-actin were measured using the real-time detection method. Total mRNA was isolated from the hippocampus, prefrontal cortex, and temporal lobe using TRIzol (Invitrogen, Carlsbad, CA, USA) and quantified by a NanoDrop 2000c system (Thermo Fisher Scientific, Waltham, MA, USA). First-strand cDNA was synthesized using a reverse transcription kit (Takara, Shiga, Japan). The sequence details of individual pairs of primers for 14-3-3β/α, haptoglobin, S100A6, ClpP, A2M, BVR-A, and β-actin are shown in Table 1. RT-PCR analysis was performed with the Thermal Cycler Dice™ SYBR green Real Time System (BioRad, CA, USA) in eight connecting tubes. The amplification conditions were as follows: initial denaturation for all pairs of primers was performed at 95°C for 3 min for one cycle, and 40 cycles of denaturation at 95°C for 5 s, annealing at 60°C for 15 s, and elongation at 60°C for 15 s were performed. The relative mRNA levels were measured by the 2^−Δ(ΔCT)^ method and normalized to the β-actin level.

**Table 1:** Primers used for 14-3-3β/α, haptoglobin, S100A6, caseinolytic protease, α-2 macroglobulin, biliverdin reductase-A, and β-actin.

**mRNA**	**Primer**	**Sequence (5'−3')**
14-3-3β/α	Sense	TGCAGTTAATACTCGGCGCT
	Antisense	GAACCACCACGGAAGCAAAC
Haptoglobin	Sense	GCCACAGACATTGAAGATGAC
	Antisense	ACTTGGGGAGTTTATCGCCAG
S100A6	Sense	TGTCGACGTGTGCTTCTAGC
	Antisense	TCACCCTCCTTGCCAGAGTA
Caseinolytic Protease	Sense	CATTCGCTGCCCAATTCCAG
	Antisense	CTCTCCATGGCTGACTCGAT
Alpha-2 macroglobulin	Sense	TGTACTCGCCAGTGCAGAAT
	Antisense	GGTACGCAGCTGGTATTCCT
Biliverdin reductase-A	Sense	GTCAACAGCTAAGTGAAGCCA
	Antisense	ATTTCCTCTTTGGCTCGGCA
β-actin	Sense	CGTTGACATCCGTAAAGACCTC
	Antisense	TAGGAGCCAGGGCAGTAATCT

### Western Blots

Western blotting was performed with primary antibodies against 14-3-3β/α (#sc25276; 1:500; Santa Cruz, CA, USA), haptoglobin (#ab256454; 1:1000; Abcam, Cambridge, UK), S100A6 (#ab181975; 1:10,000; Abcam), ClpP (#sc271284; 1:1000; Santa Cruz), A2M (#ab58703; 1:1000; Abcam), BVR-A (#sc393385; 1:1000; Santa Cruz), and β-actin (#C1313-100 1:1000; Applygen, Beijing, China) in tissues from the hippocampus, prefrontal cortex, and temporal lobe. A horseradish peroxidase conjugated labeled secondary antibody was used. Immunoreactivity was visualized by scanning the membranes with an Odyssey infrared imaging system (LI-COR). All procedures were performed by experienced experimenters blinded to the treatment condition.

### Patients and Setting

#### Ethics

The study was conducted at Beijing Jishuitan Hospital (Beijing, China) between April 2019 and August 2020. Suitable participants were at least 65 years old, suffered acute hip fracture within 3 days, had an American Society of Anesthesiologists physical status of I–III, and underwent internal fixation of hip fracture or hip replacement under general anesthesia. Written informed consent was provided by a family member or proxy; this study was registered in the Chinese Clinical Trials database (ChiCTR1900027393).

#### Study Population

**Figure 1:**
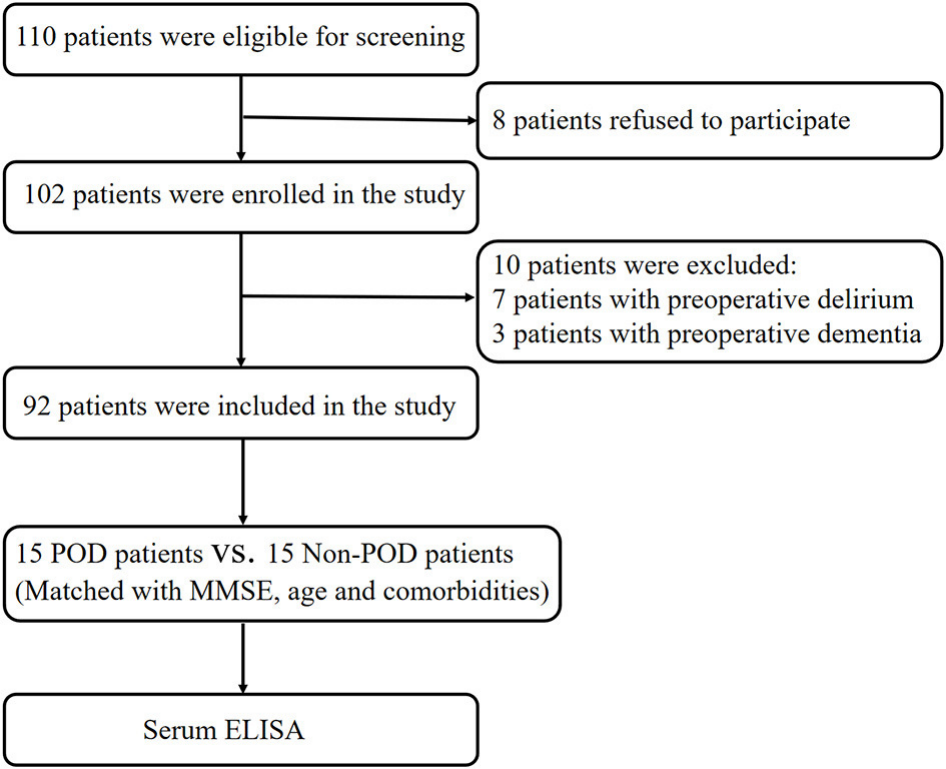
Flow diagram of the recruitment criteria. In total, 110 patients were initially screened for the study, and 30 patients were finally included in the data analysis. POD, postoperative delirium; MMSE, mini-mental state examination; ELISA, enzyme-linked immunosorbent assay.

A total of 110 eligible patients aged 65 or older with acute hip fracture injury (<72 h) who experienced POD after hip internal fixation or hip arthroplasty performed by the same surgical team under general anesthesia were enrolled in this study (Figure 1). All patients were admitted to the orthogeriatric unit. The exclusion criteria were the same as those in our previous reports (Han et al., 2020b,c) and included (1) patients with a medical history of neurological or neurovascular diseases such as delirium, schizophrenia, dementia, or stroke. Dementia was defined as a Mini-Mental State Examination (MMSE) score of ≤ 17 for illiterate patients, ≤20 for patients with 1–6 years of education, and ≤24 for patients with 7 or more years of education (Vutskits and Xie, 2016); (2) patients who were unable to read or had severe visual or auditory deficits; (3) patients with a history of alcohol abuse and drug dependence; or (4) patients who were unwilling to comply with the study protocol or procedures. Fifteen POD patients were randomly selected for quantitative proteomics studies, and 15 patients without POD were matched (1:1) to the recruited POD patients by age (±2 years) and MMSE score (±2 points). After admission, all patients received ultrasound-guided fascia iliac block to relieve pain with a single injection of 30 ml of 0.33% ropivacaine.

#### Anesthesia, Surgery, and Analgesia

All patients began fasting at 22:00 on the night before surgery. On the operation day, all patients received standard general anesthesia according to our previous report (Yuan et al., 2021). Anesthesia will be intravenously induced with 0.5–1.5 mg/kg propofol, 0.2 mg/kg etomidate, 0.3 μg/kg sufentanil and 0.15 mg/kg cisatracurium. Anesthesia will be maintained with sevoflurane, propofol and remifentanil, and the bispectral (BIS) value will be maintained between 40 and 60. A Vigileo/FloTrac device will be used for measuring stroke volume variation and cardiac index to guide fluid therapy. All operations were performed by the same group of surgeons to avoid potential confounding factors. During anesthesia, the mean arterial pressure, heart rate, BIS value, start time of anesthesia and surgery, anesthetic drugs and doses, sedative doses, blood loss, fluid balance, and transfusion of blood or clotting products, complications and adverse events, will be recorded. Moreover, patient-controlled intravenous analgesia was administered postoperatively (100 mg sufentanil and 8 mg ondansetron in 100 ml of normal saline) for 24 h as a routine analgesic regimen.

#### Neuropsychological Testing

The presence of POD was assessed using the Chinese version of the Confusion Assessment Method, which has shown good reliability and validity in elderly individuals in China (Shi et al., 2014). The Confusion Assessment Method was performed twice a day on postoperative days 1 and 2 (at 8:00 and 20:00 on each day) by a trained geriatrician.

#### Serum Collection

Preoperative venous blood (4 ml) was collected from all patients before anesthesia induction. Postoperative venous blood (4 ml) was obtained at 20:00 on postoperative day 1. After 1 h of coagulation at 4°C, blood samples were then centrifuged at 3000 × *g* at 4°C for 10 min to obtain serum. All serum samples were aliquoted and stored at −80°C for further processing.

#### Enzyme-Linked Immunosorbent Assays

Serum levels of 14-3-3β/α, haptoglobin, S100A6, ClpP, A2M, and BVR-A of both rats and human patients were determined using commercial enzyme-linked immunosorbent assay kits according to the manufacturer's instructions (Catalog No: abx500005, abx153003, abx256353, abx251576, abx155182 and abx155261, respectively; Boster, CA, US for abx500005, abx153003, abx155182 as well as abx155261 and Abbexa, Cambridge, UK for abx256353 as well as abx251576). The total protein concentration was determined using a BCA Protein Assay Kit (Beyotime, Shanghai, China). Three replicate wells were prepared for each experimental condition, and duplicate measurements were performed. The optical density was measured at a wavelength of 450 nm using a microplate reader (Varioskan Flash 3001; Thermo Fisher Scientific).

### Statistical Analysis

Data are presented as the mean ± standard error of the mean (SEM). The escape latency and swimming speed were analyzed by two-way repeated-measures analysis of variance. The results obtained from the probe test in the MWM, LC-MS/MS, PCR, western blots, and enzyme-linked immunosorbent assays were analyzed using one-way analysis of variance with multiple-comparison testing using the least significant difference test. All statistical analyses were performed using SPSS 25.0 for Windows (SPSS Inc., Chicago, IL, USA). Statistical significance was considered as *p* < 0.05.

## Results

### Surgery plus Anesthesia Resulted in Neurobehavioral Deficits in Aged Rats

**Figure 2:**
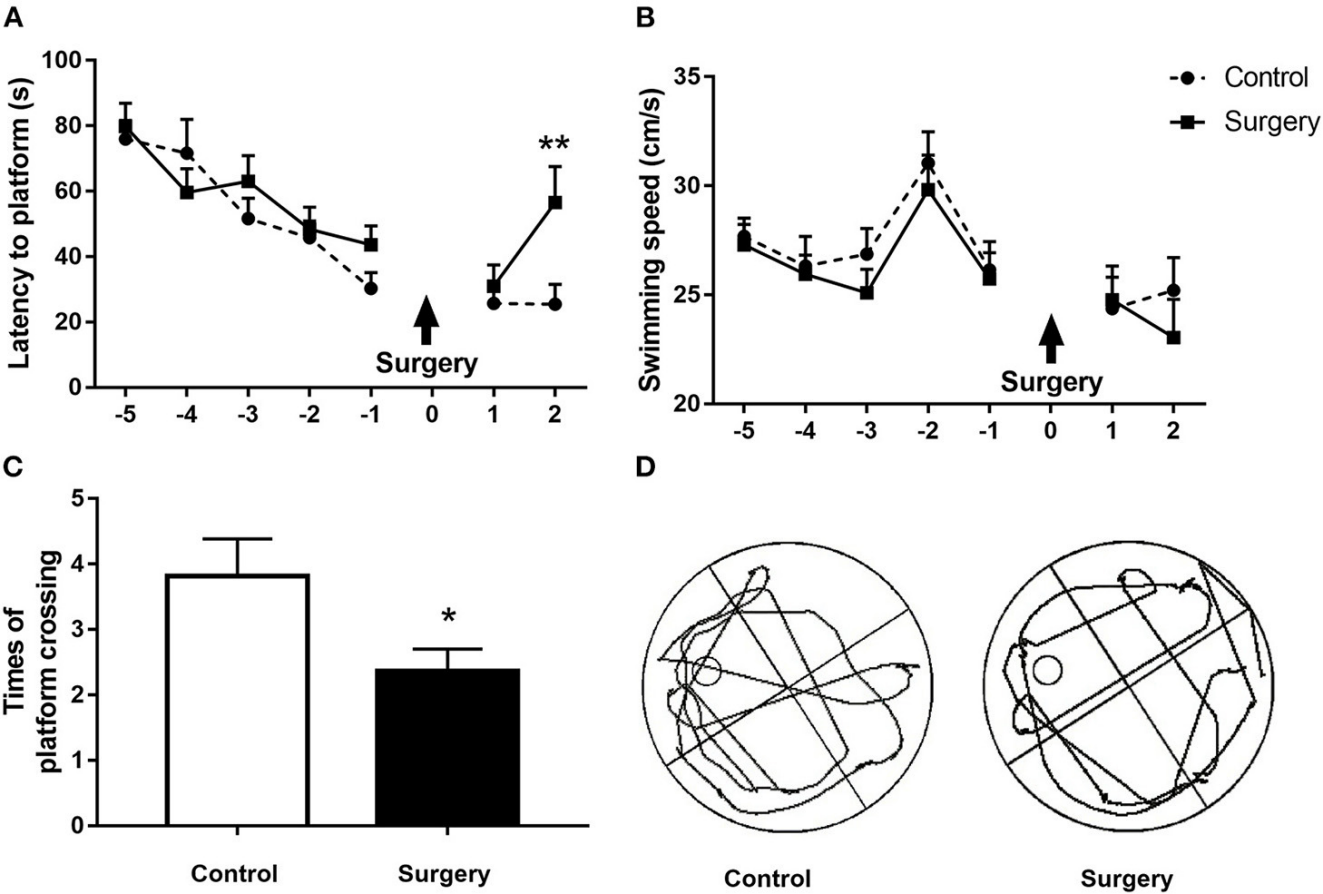
Surgery plus isoflurane anesthesia impaired spatial learning and memory in aged rats. **(A)** The surgery group had a longer escape latency on postoperative day 2 in contrast to the control group. **(B)** Anesthesia plus surgery did not alter the swimming speed during the training and probe tests. **(C)** The number of platform crossings in the surgery group was significantly decreased on postoperative day 2 compared with the control group. **(D)** The trajectory of the probe trials of the two groups on postoperative day 2. The results are presented as the mean ± SEM (*n* = 12 per group). *p < 0.05, **p < 0.01 compared with the control group.

We first used the MWM test to examine the impact of anesthesia/surgery on spatial learning and memory in aged rats. There was no significant difference between the surgery and control groups in the training step (Figure 2A, all *p* > 0.05). Animals in the surgery group displayed longer escape latencies (*p* < 0.01; Figure 2A) and fewer platform quadrant crossings (Figures 2C,D; *p* < 0.05) on the second day after surgery than the control group. The swimming speeds did not differ between the two groups during either the training or probe trials (all *p* > 0.05; Figure 2B). Our neurobehavioral results suggest that surgery plus anesthesia with endotracheal intubation, but not anesthesia alone, can cause spatial learning and memory deficits.

### Surgery plus Anesthesia Disrupted Protein Expression in the Hippocampus, Prefrontal Cortex, and Temporal Lobe

**Figure 3:**
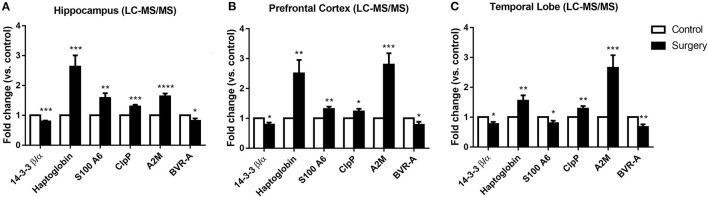
The expression of six proteins was altered in the hippocampus, prefrontal cortex, and temporal lobe of aged rats by surgery plus isoflurane according to quantitative proteomics analyses. **(A)** Surgery decreased 14-3-3β/α and biliverdin reductase-A (BVR-A) levels and increased haptoglobin, caseinolytic protease (ClpP), and α-2 macroglobulin (A2M) levels in the hippocampus. **(B)** Surgery reduced 14-3-3β/α and BVR-A levels and increased haptoglobin, S100A6, ClpP, and A2M levels in the prefrontal cortex. **(C)** Surgery decreased 14-3-3β/α, S100A6, and BVR-A levels and increased haptoglobin, ClpP, and A2M levels in the temporal lobe. The results ae presented as the mean ± SEM (*n* = 9 per group). *p < 0.05, **p < 0.01, ***p < 0.001, ****p < 0.0001 surgery vs. control group.

To explore the influence of anesthesia/surgery on the quantitative proteomics results in cognitive-related brain regions, LC-MS/MS analysis was used to detect dysregulated proteins in the hippocampus, prefrontal cortex, and temporal lobe. In total, 4,508 proteins in the hippocampus, 4,278 proteins in the prefrontal cortex, and 4,659 proteins in the temporal lobe were identified. Among them, 174 proteins in the hippocampus, 146 in the prefrontal cortex, and 119 in the temporal lobe were dysregulated. Eight proteins were commonly dysregulated in the hippocampus, prefrontal cortex, and temporal lobe following surgery plus isoflurane anesthesia according to the quantitative proteomic analysis, and among them, six proteins were expressed in both humans and rats. Among the six common affected proteins, haptoglobin, ClpP, and A2M were increased and 14-3-3β/α and BVR-A were decreased in the hippocampus, prefrontal cortex, and temporal lobe in the surgery group (*p* < 0.05, *p* < 0.01, or *p* < 0.001; Figures 3A–C). S100A6 was increased in the hippocampus and prefrontal cortex (all *p* < 0.01; Figures 3A,B) but decreased in the temporal lobe (*p* < 0.05; Figure 3C) (**Table 3**).

### Surgery plus Anesthesia Affected mRNA Expression of 14-3-3β/α, haptoglobin, S100A6, ClpP, A2M, and BVR-A

**Figure 4:**
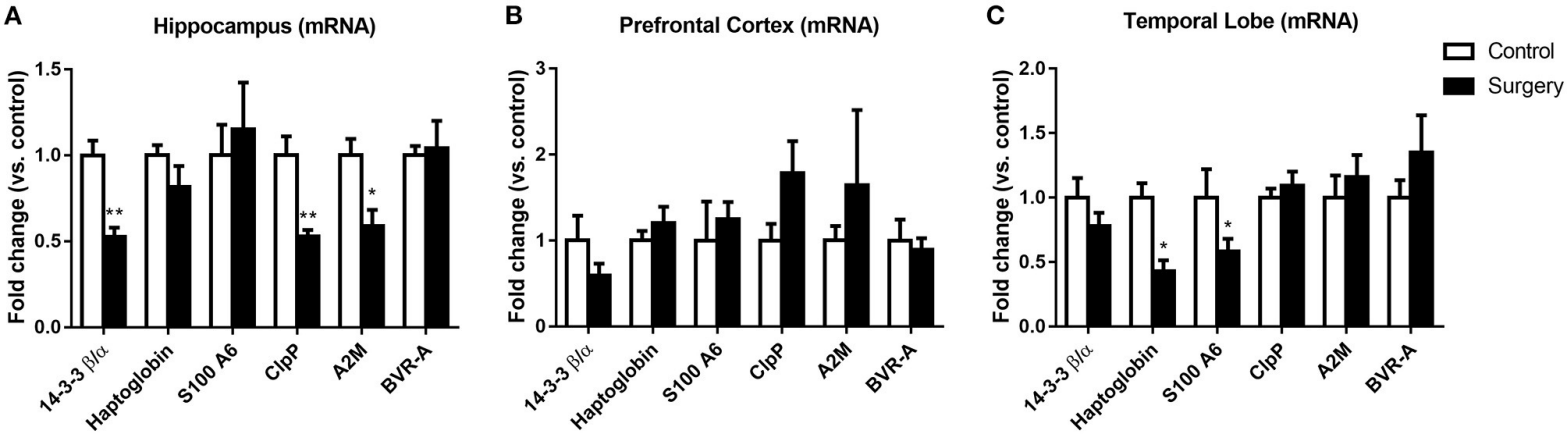
Effect of surgery plus isoflurane anesthesia on 14-3-3β/α, haptoglobin, S100A6, caseinolytic protease (ClpP), α-2 macroglobulin (A2M), and biliverdin reductase-A (BVR-A) mRNA expression in the hippocampus, prefrontal cortex, and temporal lobe of aged rats. Real-time PCR was performed on tissues from the hippocampus, prefrontal cortex, and temporal lobe. **(A)** Surgical stress reduced 14-3-3β/α, ClpP, and A2M mRNA abundance in the hippocampus. **(B)** Surgical stress did not alter the mRNA levels of the common dysregulated proteins in the prefrontal cortex. **(C)** Surgical stress decreased haptoglobin and S100A6 mRNA levels in the temporal lobe. Values represent the mean ± SEM (*n* = 5 in each group). *p < 0.05, **p < 0.01 compared with control rats.

After anesthesia and surgery, the mRNA expression levels of 14-3-3β/α, ClpP, and A2M were significantly decreased in the hippocampus (*p* < 0.05 or *p* < 0.01; Figure 4A). Moreover, the haptoglobin and S100A6 mRNA expression levels in the temporal lobe were lower in the surgery group than in the control group (all *p* < 0.05; Figure 4C). However, no significant changes in mRNA expression of the six proteins were observed in the prefrontal cortex (all *p* > 0.05; Figure 4B) (**Table 3**).

### Surgery plus Anesthesia Dysregulated Haptoglobin, S100A6, ClpP, BVR-A, A2M, and 14-3-3β/α Expression

**Figure 5:**
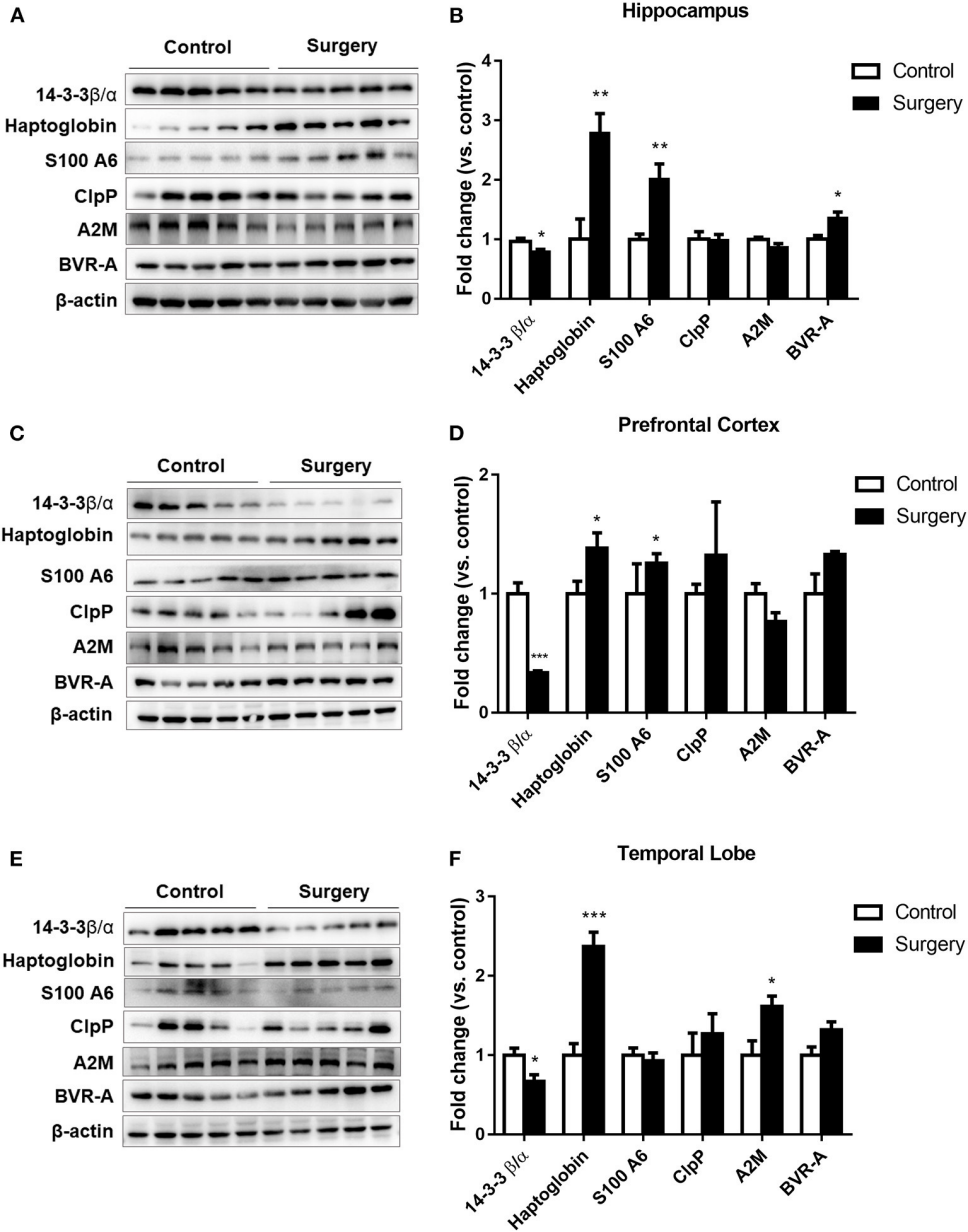
The effects of isoflurane anesthesia and surgery on the common altered proteins in aged rats. **(A)** Representative immunoblots illustrating the common affected proteins in the hippocampus after surgery. **(B)** Sevoflurane anesthesia plus surgery reduced 14-3-3β/α and increased haptoglobin, S100A6, and biliverdin reductase-A expression levels in the hippocampus. **(C**) Representative immunoblots illustrating the common altered proteins in the prefrontal cortex after surgery. **(D)** Sevoflurane anesthesia plus surgery reduced 14-3-3β/α and increased haptoglobin and S100A6 expression levels in the prefrontal cortex. **(E**) Representative immunoblots illustrating the common altered proteins in the temporal lobe after surgery. **(F)** Sevoflurane anesthesia plus surgery decreased 14-3-3β/α and increased haptoglobin and α-2 macroglobulin expression levels in the temporal lobe. Values are expressed as the mean ± SEM (*n* = 5 per group). *p < 0.05, **p < 0.01, and ***p < 0.001 surgery versus control rats.

To further verify the six common proteins altered after surgery and anesthesia exposure, we measured protein expression by western blotting. The results showed that the 14-3-3β/α level was decreased and the haptoglobin, S100A6, and BVR-A protein levels were increased in the hippocampus (*p* < 0.05 or *p* < 0.01; Figures 5A,B). Furthermore, the 14-3-3β/α level was significantly decreased and haptoglobin and S100A6 expression levels were significantly increased in the prefrontal cortex in the surgery group compared with the control group (*p* < 0.05 or *p* < 0.001; Figures 5C,D). Moreover, the 14-3-3β/α level was decreased and both the haptoglobin and A2M protein levels were increased in the temporal lobe in the surgery group (*p* < 0.05 or *p* < 0.001; Figures 5E,F) (**Table 3**).

### Characteristics of Human Participants

In this study, 15 serum samples were collected from patients with Confusion Assessment Method-confirmed POD, and 15 matched serum samples were collected from patients without POD. There were no significant differences between the POD and non-POD groups in demographic data, operative data, and comorbidities, suggesting that the case matching was successful (Table 2).

**Table 2:** Characteristics of human participants.

	**Non-POD patients (*n* = 15)**	**POD patients (*n* = 15)**	**Statistical test**	***P* value**
**Demographic data**				
Age (years)	75.1 ± 1.6	77.8 ± 2.1	t = 1.006	0.323
Male, *n* (%)	4 (27.7%)	2 (13.3%)	χ^2^ = 0.833	0.651
Height (cm)	162.2 ± 2.1	159.7 ± 1.3	t = 1.021	0.316
Weight (kg) BMI (kg/cm^2^) Education (years) Preoperative VAS score MMSE score Blood glucose (mmol/L)	62.3 ± 2.6 23.5 ± 0.6 10.6 ± 1.2 2.7 ± 0.5 26.9 ± 0.5 8.5 ± 0.7	60.1 ± 3.1 23.4 ± 0.9 8.4 ± 1.1 3.1 ± 0.5 25.4 ± 0.9 9.2 ± 0.9	t = 0.534 t = 0.141 t = 1.407 t = −0.578 t = 1.391 t = −0.573	0.598 0.889 0.170 0.568 0.177 0.571
**Operative data**				
ASA class, *n* (%)			χ^2^ = 0.833	0.651
II III	13 (86.7) 2 (13.3)	11 (73.3) 4 (26.7)		
Length of anesthesia (min)	82.3 ± 7.1	81.7 ± 7.7	t = 0.057	0.955
Length of surgery (min)	37.7 ± 5.0	44.7 ± 6.8	t = −0.826	0.416
**Comorbidities**				
Coronary heart disease, *n* (%)	3 (20.0)	4 (26.7)	χ^2^ = 0.186	0.666
Cerebral infarction, *n* (%)	2 (13.3)	2 (13.3)	χ^2^ = 0.000	1
Hypertension, *n* (%)	11 (73.3)	12 (80.0)	χ^2^ = 0.186	0.666
Diabetes, *n* (%)	9 (60.0)	9 (60.0)	χ^2^ = 0.000	1

*POD, Postoperative Delirium; BMI, Body Mass Index; VAS, Visual Analog Scale; MMSE, Mini-Mental State Examination; ASA, American Society of Anesthesiologists*.

### Surgery plus Anesthesia Perturbed 14-3-3β/α, A2M, and ClpP Expression in the Serum of Both Aged dNCR Rats and Older Patients With POD

To further explore the potential of these proteins as convenient diagnostic biomarkers for po-NCD, we investigated changes in these proteins in the serum of aged dNCR rats and elderly POD patients.

**Figure 6:**
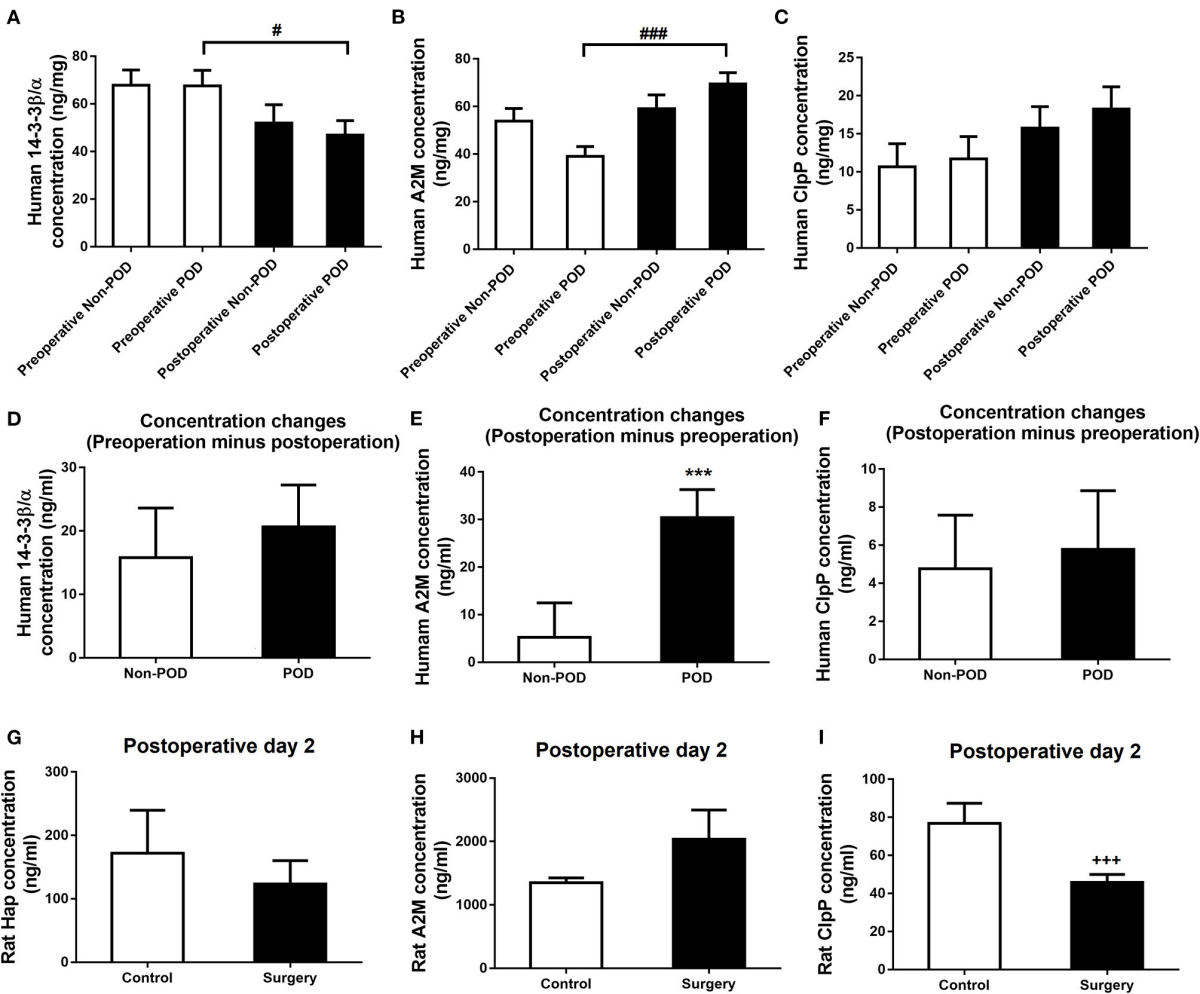
Human and rat serum levels of the common altered proteins. **(A, B)** Decreased human serum 14-3-3β/α and increased α-2 macroglobulin (A2M) levels were found postoperatively in elderly postoperative delirium (POD) patients compared with the preoperative levels, but no significant human serum ClpP concentration was observed **(C)**. **(D)** The change in the postoperative minus preoperative serum 14-3-3β/α concentration was not significant in POD than in non-POD patients. **(E)** The change in the postoperative minus preoperative serum A2M concentration was significantly higher in POD than in non-POD patients. **(F)** The change in the postoperative minus preoperative serum ClpP concentration was not significant in POD than in non-POD patients. **(G, H)** Rat serum haptoglobin and A2M levels was also significantly changed in the surgery group compared with control rats. **(I)** Rat serum caseinolytic protease was significantly reduced in the surgery group compared with control rats. Values represent mean ± SEM (*n* = 15 humans or *n* = 8 rats in each group). ^#^*p* < 0.05, ^*###*^*p* < 0.001 postoperative human serum concentration compared with the preoperative concentration, ***p < 0.001 POD vs. non-POD patients, ^**+++**^*p* < 0.001 surgery vs. control rats.

The serum concentrations of 14-3-3β/α and A2M in postoperative POD patients were significantly higher than those in preoperative POD patients (*p* < 0.05 or *p* < 0.001, respectively, Figures 6A,B), but there was no difference in ClpP (*p* > 0.05; Figure 6C). The difference between the postoperative and preoperative serum concentration of A2M was significantly increased in POD patients in contrast to non-POD controls (*p* < 0.001; Figure 6E), but not 14-3-3β/α and ClpP (*p* > 0.05; Figures 6D,F). Furthermore, the difference in postoperative and preoperative serum concentrations of ClpP was significantly decreased in aged dNCR rats compared with control rats (*p* < 0.001; Figure 6I), but there were no significant difference in Hap and A2M (*p* > 0.05; Figures 6G,H) (Table 3).

**Table 3:** Effects of anesthesia/surgery on the common dysregulated proteins in both aged dNCR rats and elderly POD patients.

**Gene Symbol**	**Protein**	**HP**			**PFC**			**TC**			**Rat serum**	**Humanserum**
		**MS**	**PCR**	**WB**	**MS**	**PCR**	**WB**	**MS**	**PCR**	**WB**		
*Clpp*	ATP-dependent Clp protease proteolytic subunit	↑*↑↑*	↓**↓**	—	↑	—	—	↑↑	—	—	↓*↓↓*	—
*S100a6*	S100 A6	↑↑	—	↑↑	↑↑	—	↑	↓	↓	—	—	—
*A2m*	Alpha-2-macroglobulin	↑*↑↑↑*	↓	—	↑*↑↑*	—	—	↑*↑↑*	—	↑	—	↑*↑↑*
*Hp*	Haptoglobin	↑*↑↑*	—	↑↑	↑↑	—	↑	↑↑	↓	↑*↑↑*	—	—
*Ywhab*	14-3-3 protein beta/alpha	↓*↓↓*	↓**↓**	↓	↓	—	↓*↓↓*	↓	—	↓	—	↓
*Blvra*	Biliverdin reductase A	↓	—	↑	↓	—	—	↓↓	—	—	—	—

***↑**, ↑↑, ↑↑↑, and ↑↑↑**↑** indicate a significant increase (p <0.05, p < 0.01, p <0.001, and p < 0.0001, respectively) in the anesthesia/surgery condition compared with the control condition including both the animal and clinical investigations. Moreover, **↓**, ↓**↓**, and ↓↓↓ indicate a significant decrease (p < 0.05, p < 0.01, and p < 0.001, respectively) in the anesthesia/surgery condition compared with the control condition including both the animal and clinical investigations*.

*The increase in serum α-2 macroglobulin and decrease in serum 14-3-3β/α in geriatric postoperative delirium patients as well as the decrease in serum caseinolytic protease (ClpP) in aged delayed neurocognitive recovery rats suggest their potential as diagnostic biomarkers of postoperative neurocognitive disorders*.

## Discussion

In the present study, we explored commonly altered proteins and their changes using proteomics and molecular biological methods, respectively, in the hippocampus, prefrontal cortex, and temporal lobe of aged dNCR rats. We found that haptoglobin, ClpP, and A2M expression levels were increased and 14-3-3β/α and BVR-A expression levels were decreased in the hippocampus, prefrontal cortex, and temporal lobe according to quantitative proteomics methods. Moreover, decreased serum 14-3-3β/α and increased A2M levels in geriatric POD patients as well as decreased serum ClpP levels in aged dNCR rats were also observed, suggesting that serum 14-3-3β/α, A2M, and ClpP levels may have translational potential for clinical prediction of po-NCD.

Our research team has investigated POD biomarkers in the CSF of elderly POD patients using metabolomics and proteomics (Han et al., 2020b,c). However, in contrast to peripheral blood sampling, CSF availability is limited. CSF sampling cannot be performed in all patients or at any time point because of patient discomfort and safety reasons. Therefore, serum sampling was used in this study as a means to more conveniently preliminarily examine potential biomarkers for po-NCD.

Currently, it is still uncertain whether POD and dNCR are distinct pathologies or “two sides of the same coin” (Olotu, 2020), mainly because of the lack of animal models of POD. Peng et al. used 4-month-old mice to determine the effects of anesthesia/surgery on natural and learned behaviors, and the results suggested that this mouse model could exhibit acute onset and a fluctuating course of behaviors similar to POD (Peng et al., 2016; Chen et al., 2021). Illendula et al. used 18- to 20-month-old mice to examine anesthesia, surgery, and the intensive care unit environment and found that these insults impaired mouse behaviors that depend on attention, memory, and thought organization. The changes also showed acute onset and fluctuated over time (Illendula et al., 2020). Our research group also used a battery of behavioral tests to assess the effects of laparotomy under isoflurane anesthesia in aged mice and found that inattention, the core symptom of delirium, occurred at 6 or 9 h and recovered at 24 h after anesthesia/surgery (Liu et al., 2020). We also demonstrated that surgery plus isoflurane anesthesia could induce delirium-like neurobehavioral deficits in aged rats (data not shown). Moreover, a pathognomonic feature of POD is inattention, which may be mediated by prefrontal cortex dysfunction (Culley et al., 2014; Bahmani et al., 2019), and we also found pathological changes in the prefrontal cortex in our dNCR rat model. Therefore, this animal model could be used to partially elucidate the pathophysiological mechanisms of short-term po-NCD including POD and recent dNCR.

The 14-3-3 proteins comprise a family of seven conserved proteins that are highly expressed in the brain. These proteins are critical for neuronal function because they affect protein folding, trafficking, and neurite growth (Berg et al., 2003; Mrowiec and Schwappach, 2006; Kajiwara et al., 2009; Sluchanko and Gusev, 2017). The 14-3-3 proteins can also reduce cell-to-cell transfer and propagation of α-synuclein, a key pathogenic protein in Parkinson's disease (Wang et al., 2018), and can exert a neuroprotective effect against α-synuclein toxicity *in vivo* (Underwood et al., 2021). Moreover, α-synuclein overexpression can also repress 14-3-3 protein transcription (Ding et al., 2013). Our previous findings also indicated that α-synuclein oligomerization in the hippocampus may be an important mechanism in dNCR (Yang et al., 2020), which is accordance with the decreased 14-3-3β/α expression in aged dNCR rats found in the present study. More interestingly, serum 14-3-3β/α was also decreased in postoperative POD patients. However, no studies have investigated the specific mechanism of 14-3-3β/α and how it passes through the blood brain barrier into the blood in po-NCD.

The acute-phase protein A2M is present in body fluids of both invertebrates and vertebrates and is a major component of the innate immune system, functioning as a pan-protease inhibitor and chaperone protein (Cater et al., 2019). A2M can stabilize misfolded proteins, prevent their aberrant aggregation, and facilitate their clearance, as shown for the Alzheimer's disease-associated amyloid β peptide and Parkinson's disease-associated α-synuclein (Wyatt et al., 2013a,b; Whiten et al., 2018). In this study, A2M protein was significantly elevated in the temporal lobe of aged dNCR rats. We speculate that this may be a protective response to the abnormal accumulation of amyloid β and α-synuclein, which may be an important pathophysiological mechanism of po-NCD (Dong et al., 2009; Yang et al., 2020). Moreover, the difference in the postoperative and preoperative serum A2M concentration was significantly higher in POD patients than in non-POD patients. The mechanism by which A2M crosses the blood-brain barrier and enters the peripheral blood after anesthesia/surgery is worthy of further exploration.

ClpP is an energy-dependent serine protease that is highly conserved from bacteria to eukaryotic mitochondria and chloroplasts (Yu and Houry, 2007). It plays a vital role in protein quality control within the mitochondrial matrix and maintains protein homeostasis (Kress et al., 2009). Currently, the relationship between ClpP and po-NCD has not been investigated. In this study, anesthesia/surgery significantly decreased ClpP mRNA expression in the hippocampus but did not affect ClpP protein expression. Interestingly, the serum ClpP concentration was significantly decreased in aged dNCR rats compared with controls. Because mitochondrial dysfunction is an important pathophysiological mechanism of dNCR (Li et al., 2017; Netto et al., 2018), we speculate that ClpP that exhibits normal physiological function can penetrate the blood brain barrier, and this function decreases in aged dNCR rats.

Haptoglobin is an acute phase protein produced by the liver and abundant in the plasma of humans and other mammals (Sadrzadeh and Bozorgmehr, 2004). Under normal physiological conditions, haptoglobin can combine with free hemoglobin to form a haptoglobin-hemoglobin complex that can eliminate cell-free hemoglobin and its byproducts by binding with the scavenger receptor CD163, inducing the same anti-oxidative and anti-inflammatory effects as free hemoglobin (Kristiansen et al., 2001). Haptoglobin is also induced and released in the peripheral blood and acts as a marker of inflammation (Bowman and Kurosky, 1982). Moreover, Song et al. found that compared with healthy controls, patients with Alzheimer's disease had significantly higher mean serum haptoglobin levels (Song et al., 2015). In addition, Zhu et al. further found that elevated serum levels of haptoglobin were observed both in Alzheimer's disease and mild cognitive impairment patients, and the haptoglobin level may be correlated with the severity of Alzheimer's disease (Zhu et al., 2018). In our recent study, haptoglobin was elevated in three cognitive-related brain regions, the hippocampus, prefrontal cortex, and temporal lobe, indicating that anesthesia/surgery induced inflammation and oxidative stress in these brain regions of aged rats.

S100A6 is a Ca^2+^/Zn^2+^-binding protein and belongs to the large S100 protein family (Lesniak et al., 2017). S100A6 monomer binds two Ca^2+^ ions and then induces conformational changes to induce interactions with target proteins and transduction of Ca^2+^ signals. Under physiological conditions, S100A6 is mainly expressed in neurons in the amygdala and entorhinal cortex and in some astrocytes (Yamashita et al., 1999). Intriguingly, S100A6 overexpression in astrocytes has been observed near amyloid plaque deposits in Alzheimer's disease patients and Alzheimer's disease transgenic mouse models (Boom et al., 2004). Amyloid is also considered to be involved in the pathophysiological process of dNCR (Dong et al., 2009). In this study, S100A6 protein expression was significantly increased in the hippocampus and prefrontal cortex of the surgery group compared with the control rats. We speculate that this may be a protective mechanism. The mechanism by which S100A6 penetrates the blood brain barrier and enters the peripheral blood after anesthesia/surgery is worthy of further exploration. BVR-A is a pleiotropic enzyme that plays a pivotal role in the antioxidant defense against free radicals and in cell homeostasis (Kapitulnik and Maines, 2009). Sharma et al. used a 3xTg-AD mouse model and found that early reduction of BVR-A protein levels is associated with reduced GSK-3β inhibition in the hippocampus, which is a key early event promoting Tau phosphorylation in Alzheimer's disease (Rockenstein et al., 2007; Sharma et al., 2019). There is also reported that increased BVR-A decreased oxidative stress levels in parietal cortex and improved cognitive functions in a dog-preclinical model of Alzheimer disease (Barone et al., 2012), implying that BVR-A contributes to the neuroprotective effect. In the present study, we found that BVR-A in hippocampus was significantly lower by LC-MS/MS and significantly higher by western blot. We speculate that there may be two reasons. First, the sample size of each group was 9 in LC-MS/MS and 5 in western blot. Second, LC-MS/MS analysis which belongs to tandem mass spectrometry separates and detects charged ions according to the mass charge ratio under the action of electric field. It can accurately analyze compounds qualitatively and quantitatively (Aslam et al., 2017; Andries et al., 2021). The basic principle of western blot is the specific binding of antigen and antibody, which impossible to accurately determine the protein expression level (Kurien and Scofield, 2006; Aslam et al., 2017). So we conjecture that the LC-MS/MS analysis may be more credible. Given that BVR-A contributes to the neuroprotective effect, decreased BVR-A proteins weakens the neuroprotective effect which is consistent with the results of BVR-A LC-MS/MS in our recent study. However, the pathophysiological mechanism of BVR-A in po-NCD has still not been reported.

There are several limitations to the current study. First, we used the established dNCR model with endotracheal intubation anesthesia according to our previous study (Li et al., 2016; Mi et al., 2021). Compared with other dNCR models, this model is more clinically relevant. However, although isoflurane has some analgesic and muscle relaxant effects, and we also used oxybuprocaine hydrochloride gel to relieve pain, these drugs cannot completely replace muscle relaxants and opioid analgesics during anesthesia. The effects of analgesia and muscle relaxation deficiency on the experimental results need to be further evaluated. Second, we only focused on the hippocampus, prefrontal cortex, and temporal lobe in this study. Other forebrain regions such as the amygdala, parietal cortex, occipital cortex, temporal cortex, and striatum have been implicated in cognition and attention, but they were not investigated in this study. Third, there is a lack of parallelism between mRNA and protein levels in some proteins. Gene expression is multistep biochemical process, involving transcription, translation and subsequent protein modification (Raj and van Oudenaarden, 2008). From this process, genetic information from the archival copy of DNA to the short-lived mRNA, and ultimately to specific proteins (Gibney and Nolan, 2010). The lack of parallelism between mRNA and protein levels in the present study may be partially because these proteins experienced complexed and distinct post-translational modification processes. However, we did not explore what kinds of post-translational modifications these proteins undergone. Nevertheless, this study demonstrated that decreased serum 14-3-3β/α and ClpP levels as well as an increased A2M level may be promising diagnostic biomarkers for short-term po-NCD. These novel altered serum proteins are likely to be involved in po-NCD. Our results not only provide potential risk biomarkers for po-NCD but also provide valuable information for further pathological mechanism studies.

## Data Availability Statement

The datasets presented in this study can be found in online repositories. The names of the repository/repositories and accession number(s) can be found at: ProteomeXchange [accession: PDX028045].

## Ethics Statement

The studies involving human participants were reviewed and approved by Chinese Clinical Trials database. The patients/participants provided their written informed consent to participate in this study. The animal study was reviewed and approved by Peking University Biomedical Ethics Committee Experimental Animal Ethics Branch. Written informed consent was obtained from the owners for the participation of their animals in this study.

## Author Contributions

NY and XG: conceived and designed this project. YTL, LC, ZL, YS, YY, TL, JHo, ZK, JHe, YL, and XM: performed the experiments. YTL: generated the figures. YTL and LC: conducted the statistical analyses and wrote the first draft of the manuscript. HC and QW: contributed to the revised manuscript. NY and XG: modified the manuscript. All authors read and approved the final manuscript.

## Funding

This work was supported by the National Natural Science Foundation of China (81901095, 82071189, 81873726, and 81971012), the Interdisciplinary Medicine Seed Fund of Peking University (BMU2021MX026), and Peking University Clinical Medicine plus X Youth Project (PKU2020LCXQ016).

## Conflict of Interest

The authors declare that the research was conducted in the absence of any commercial or financial relationships that could be construed as a potential conflict of interest.

## Publisher's Note

All claims expressed in this article are solely those of the authors and do not necessarily represent those of their affiliated organizations, or those of the publisher, the editors and the reviewers. Any product that may be evaluated in this article, or claim that may be made by its manufacturer, is not guaranteed or endorsed by the publisher.
