# Supplementary figures and images for: Potential Serum Biomarkers for Postoperative Neurocognitive Disorders Based on Proteomic Analysis of Cognitive-Related Brain Regions
Source: Front Aging Neurosci. 2021 Sep 29;13:741263. doi: 10.3389/fnagi.2021.741263 (PMC8511679; doi:10.3389/fnagi.2021.741263)

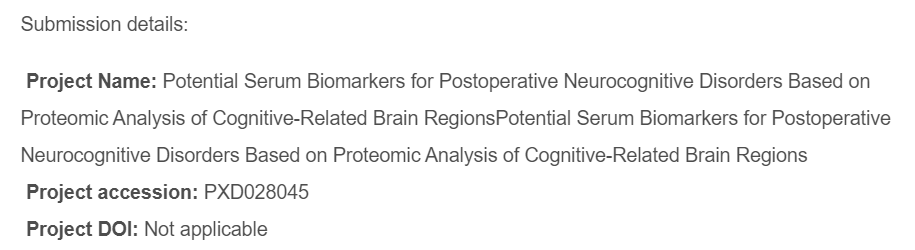

Supplement: Supplementary file 1 [file Data_Sheet_1.ZIP › Source Data/MS accession number/feb4625b4d17ae276db996555888b3a.png]

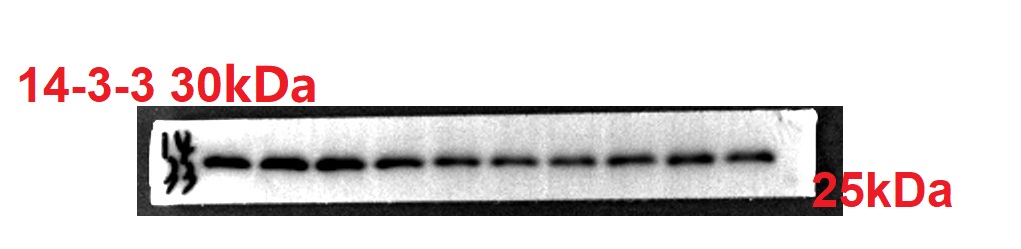

Supplement: Supplementary file 1 [file Data_Sheet_1.ZIP › Source Data/Western blot/hippoocampus/14-3-3.jpg]

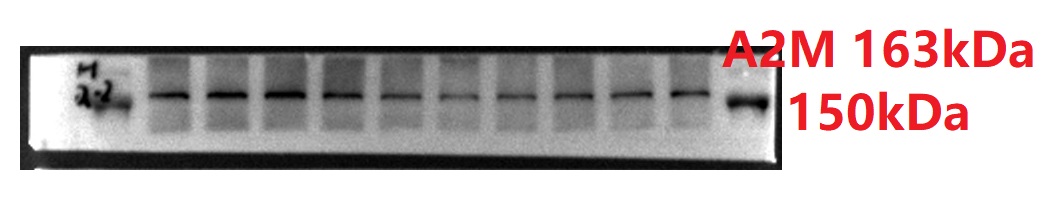

Supplement: Supplementary file 1 [file Data_Sheet_1.ZIP › Source Data/Western blot/hippoocampus/A2M.jpg]

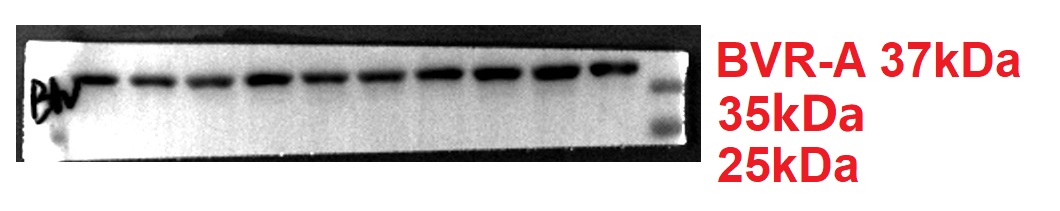

Supplement: Supplementary file 1 [file Data_Sheet_1.ZIP › Source Data/Western blot/hippoocampus/BVR-A.jpg]

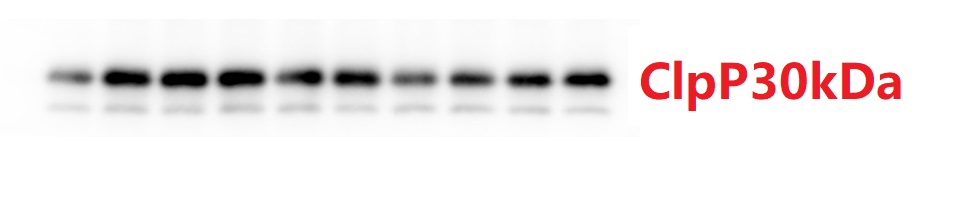

Supplement: Supplementary file 1 [file Data_Sheet_1.ZIP › Source Data/Western blot/hippoocampus/Clpp.jpg]

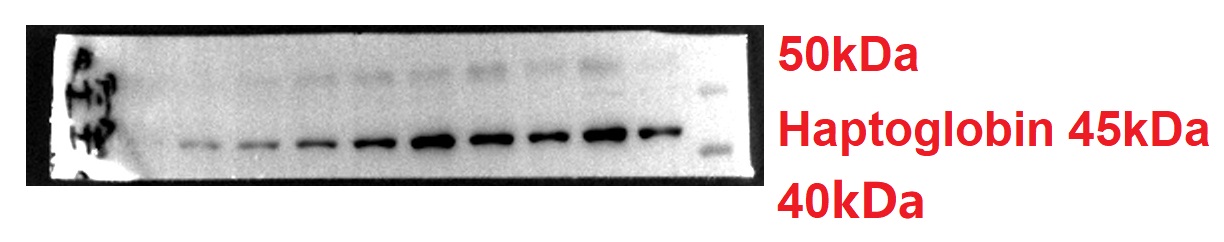

Supplement: Supplementary file 1 [file Data_Sheet_1.ZIP › Source Data/Western blot/hippoocampus/Haptoglobin.jpg]

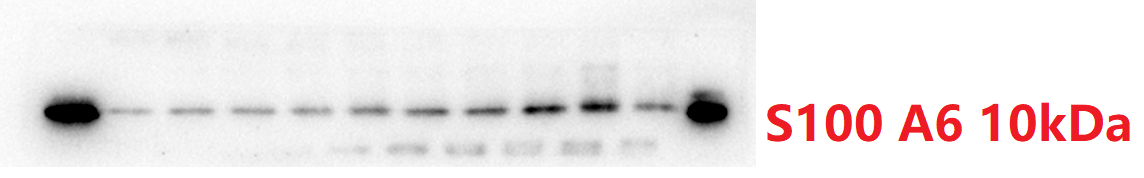

Supplement: Supplementary file 1 [file Data_Sheet_1.ZIP › Source Data/Western blot/hippoocampus/S100.Tif]

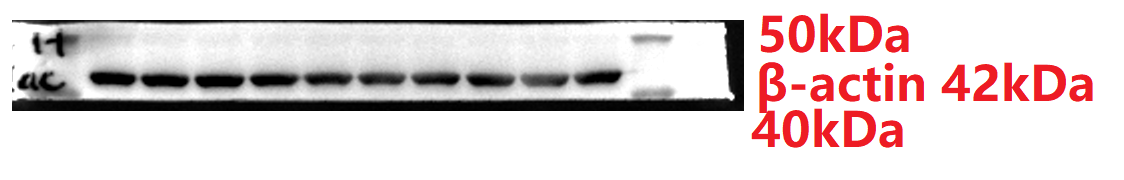

Supplement: Supplementary file 1 [file Data_Sheet_1.ZIP › Source Data/Western blot/hippoocampus/actin.Tif]

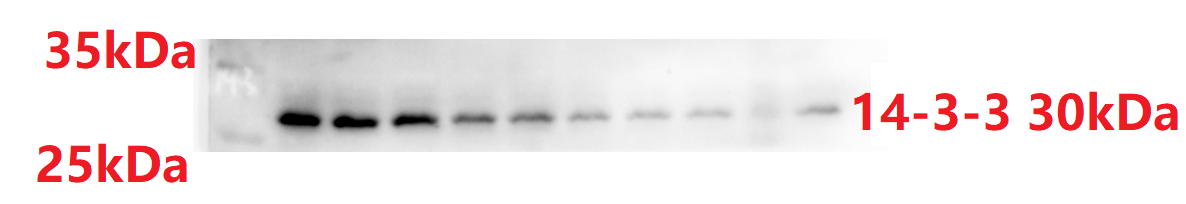

Supplement: Supplementary file 1 [file Data_Sheet_1.ZIP › Source Data/Western blot/prefrontal cortex/14-3-3.Tif]

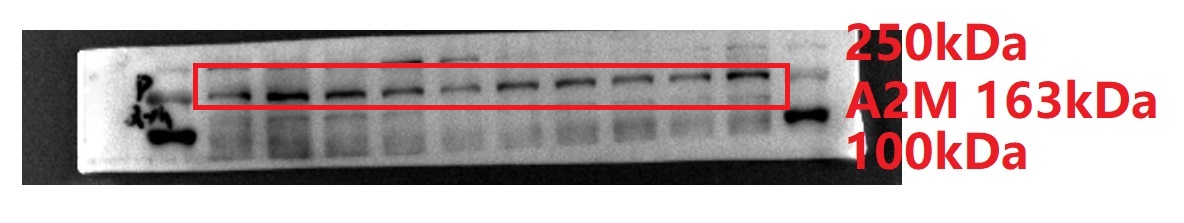

Supplement: Supplementary file 1 [file Data_Sheet_1.ZIP › Source Data/Western blot/prefrontal cortex/A2M.jpg]

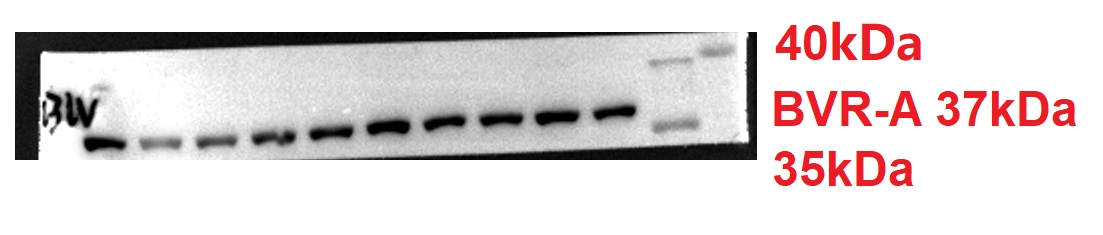

Supplement: Supplementary file 1 [file Data_Sheet_1.ZIP › Source Data/Western blot/prefrontal cortex/BVR-A.jpg]

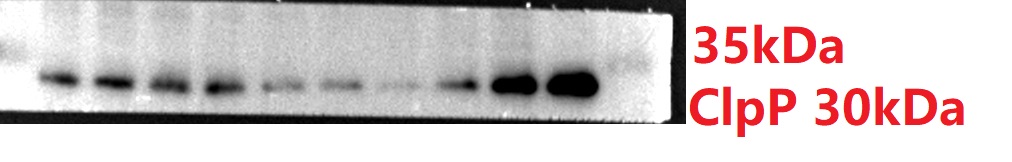

Supplement: Supplementary file 1 [file Data_Sheet_1.ZIP › Source Data/Western blot/prefrontal cortex/CLPP.jpg]

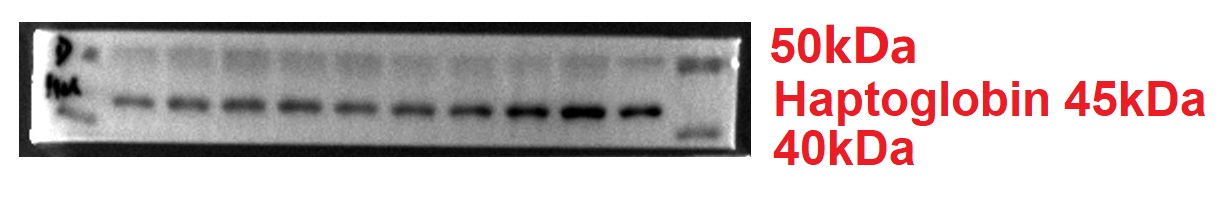

Supplement: Supplementary file 1 [file Data_Sheet_1.ZIP › Source Data/Western blot/prefrontal cortex/Haptoglobin.jpg]

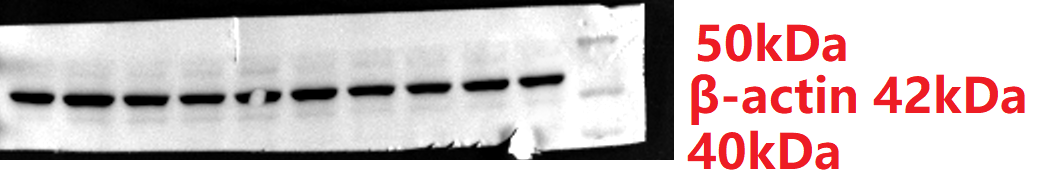

Supplement: Supplementary file 1 [file Data_Sheet_1.ZIP › Source Data/Western blot/prefrontal cortex/actin.Tif]

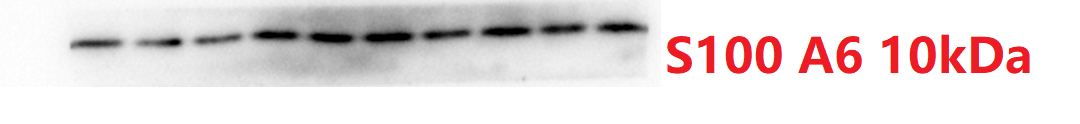

Supplement: Supplementary file 1 [file Data_Sheet_1.ZIP › Source Data/Western blot/prefrontal cortex/s100.tif]

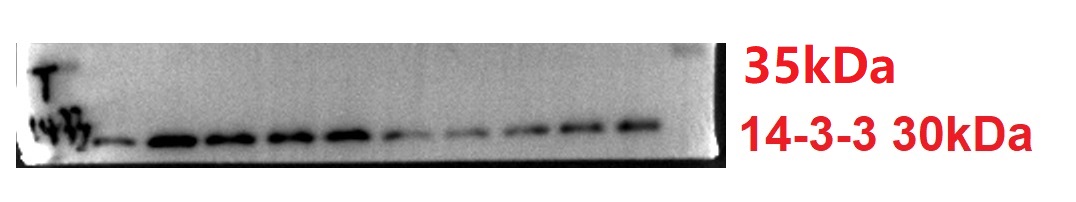

Supplement: Supplementary file 1 [file Data_Sheet_1.ZIP › Source Data/Western blot/temporal lobe/14-3-3.jpg]

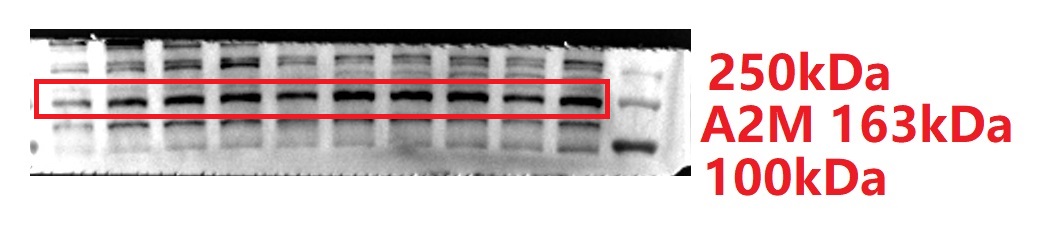

Supplement: Supplementary file 1 [file Data_Sheet_1.ZIP › Source Data/Western blot/temporal lobe/A2M.jpg]

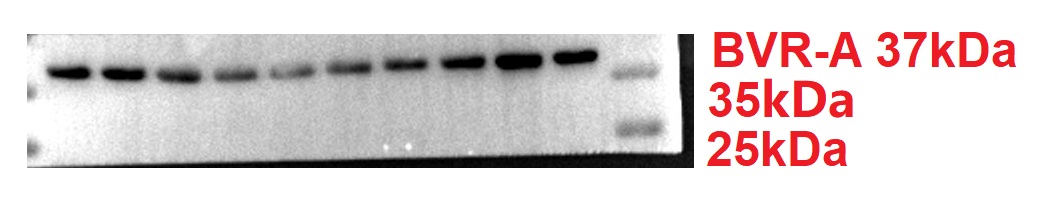

Supplement: Supplementary file 1 [file Data_Sheet_1.ZIP › Source Data/Western blot/temporal lobe/BVR-A.jpg]

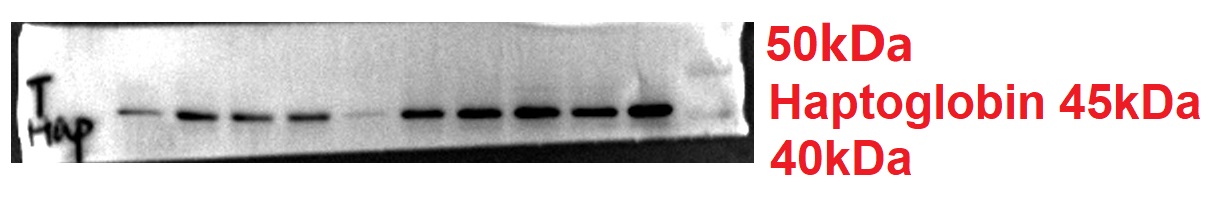

Supplement: Supplementary file 1 [file Data_Sheet_1.ZIP › Source Data/Western blot/temporal lobe/Haptoglobin.jpg]

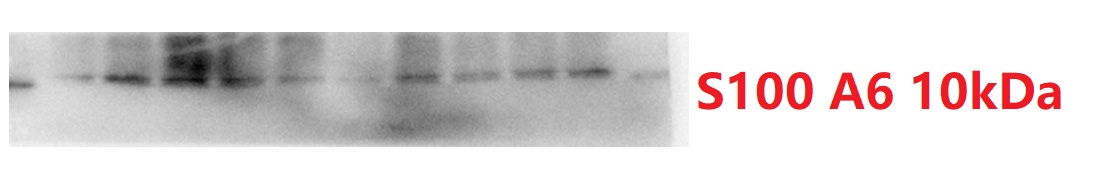

Supplement: Supplementary file 1 [file Data_Sheet_1.ZIP › Source Data/Western blot/temporal lobe/S100.jpg]

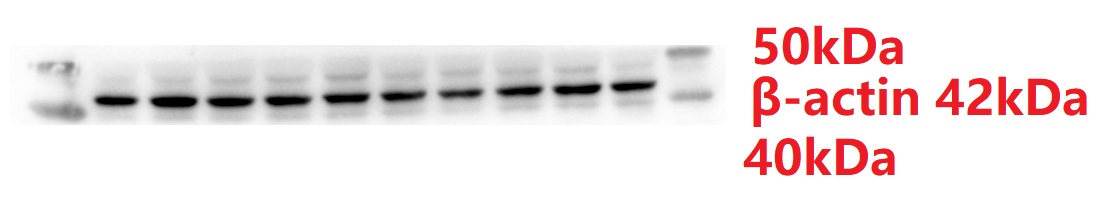

Supplement: Supplementary file 1 [file Data_Sheet_1.ZIP › Source Data/Western blot/temporal lobe/actin.jpg]

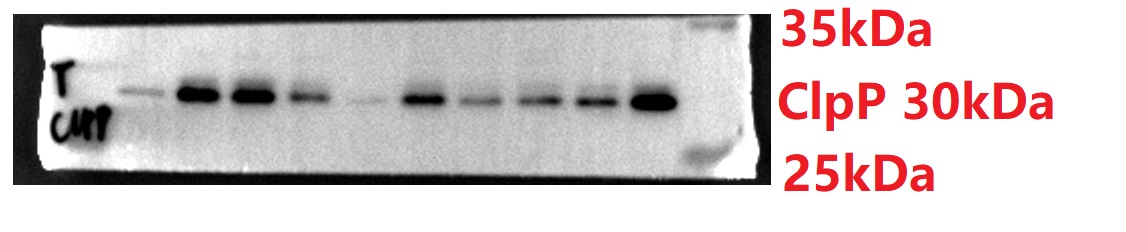

Supplement: Supplementary file 1 [file Data_Sheet_1.ZIP › Source Data/Western blot/temporal lobe/clpp.jpg]
